# Immunomodulation and infection: identification of small molecule TLR3 blockers to combat deleterious inflammation in pneumonia

**DOI:** 10.1186/cc14058

**Published:** 2014-12-03

**Authors:** F Sônego, B Pouzet, F Pecceu, L Leotard, B Martin, P-E Rouby, L Goullieux, C Ravinet-Trillou, J-M Bras, C Serradeil-Le Gal

**Affiliations:** 1 Sanofi TSU ID, Toulouse, France; 2 SCP LGCR, Toulouse, France; 3 Biologics, Toulouse, France

## Introduction

Pneumonia is a common cause of death worldwide and the leading cause of sepsis and shock in the ICU. Controlling excessive immune stimulation with deleterious consequences for the host organs is a major strategy in severe infections. In addition to viral dsRNA, the immune Toll-like receptor 3 (TLR3) senses RNA released from injured tissues and necrotic cells promoting excessive inflammatory cytokine release in pulmonary epithelial, endothelial and immune cells. Thus, as a potent regulator of the lung immune response, TLR3 represents a specific target to control damaging inflammation in severe lung infections in combination with antimicrobials. Here, we aimed to characterize potent small molecules displaying *in vitro *and *in vivo *TLR3 blocker activities and develop several preclinical models to investigate the role of TLR3 in pneumonia.

## Results

We identified compounds with anti-TLR3 activity (IC_50 _= 50 nM) through random screening and structure-activity relationship analysis by testing small molecule libraries on recombinant hTLR3-HEK293 cells. Selected compounds interacted with mouse and human TLR3, were devoid of effect on TNFα-induced activation at 1 µM on HEK293 cells and exhibited good early-ADME properties. Hit molecules also counteracted PolyAU (PAU)/Poly(I:C) (PIC)-induced cytokine release (IL6, IL8, IP-10) on human bronchial epithelial cells (BEAS-2B) expressing native TLR3 receptors. Interestingly, sustained stimulation of BEAS-2B by PAU/PIC (0.1 to 10 µM) to mimic viral activation induced overexpression of TLR3 mRNA, which was dose-dependently inhibited by these compounds, as evidenced by RT-qPCR. *In vivo*, we confirmed the anti-TLR3 activity (3 to 30 mg/kg i.p.) against PAU/PIC (100 µg/mouse i.v.) but not flagellin-induced plasma cytokine release in CD1 mice. To study these TLR3 inhibitors under pathological situations, we developed models of *Streptoccocus pneumoniae *(ATCC6303) or *Pseudomonas aeruginosa *(PAO-1)-induced pneumonia post influenza A virus (IAV PR/8/34 H1N1) infection. Mice previously challenged with intranasal IAV showed enhanced susceptibility to lung bacterial infections with a dramatic mortality rate, whereas separately these pathogens are not lethal. In addition, IAV/*S. pneumoniae *co-infected mice showed increased lung and blood bacterial loads with severe inflammation signs evidenced by elevated systemic IL-6 and KC levels and important lung tissue damages. Currently, TLR3 blockers are ongoing evaluation in these models.

## Conclusion

Immunomodulation and personalized treatments are becoming relevant approaches to manage severe infections. Here, we discovered the first small molecule probes targeting mouse and human TLR3. These compounds efficiently control TLR3-triggered proinflammatory response *in vitro *and *in vivo *and shape TLR3 overexpression in human lung epithelial cells. They represent valuable pharmacological tools to study the contribution of TLR3 in pneumonia and to propose new adjuvant immunotherapies.

